# Maternal Morbidity in Women with Placenta Previa Managed with Prediction of Morbidly Adherent Placenta by Ultrasonography

**DOI:** 10.1155/2017/8318751

**Published:** 2017-04-24

**Authors:** Midori Fujisaki, Seishi Furukawa, Yohei Maki, Masanao Oohashi, Koutarou Doi, Hiroshi Sameshima

**Affiliations:** ^1^Department of Obstetrics & Gynecology, Faculty of Medicine, University of Miyazaki, Miyazaki, Japan; ^2^Department of Obstetrics & Gynecology, School of Medicine, Kyorin University, Tokyo, Japan

## Abstract

*Objective*. To determine maternal morbidity in women with placenta previa managed with prediction of morbidly adherent placenta (MAP) by ultrasonography.* Methods*. A retrospective cohort study was undertaken comprising forty-one women who had placenta previa with or without risk factors for MAP. Women who had all three findings (bladder line interruption, placental lacunae, and absence of the retroplacental clear zone) were regarded as high suspicion for MAP and underwent cesarean section followed by hysterectomy. We attempted placental removal for women having two findings or less.* Results*. Among 28 women with risk, nine with high suspicion underwent hysterectomy and were diagnosed with MAP. Three of 19 women with two findings or less eventually underwent hysterectomy and were diagnosed with MAP. The sensitivity and positive predictive value for the detection of MAP were 64% and 100%. The pathological severity of MAP was significantly correlated with the cumulative number of findings. There were no cases of MAP among 13 women without risk. There was no difference of blood loss between women with high suspicion and those without risk (2186 ± 1438 ml versus 1656 ± 848 ml, resp.; *p* = 0.34).* Conclusion*. Management with prediction of MAP by ultrasonography is useful for obtaining permissible morbidity.

## 1. Introduction

Morbidly adherent placenta (MAP) is one of a number of risk factors related to maternal death [1]. Among women with obstetrical bleeding and who subsequently receive blood transfusions, MAP accounts for 15% of all cases and is associated with severe morbidity, in addition to an increased likelihood for the use of invasive procedures such as hysterectomy (78%), massive blood loss over 3000 ml (65%), and massive blood transfusion with ≥10 units of packed red blood cells and/or ≥10 units of fresh frozen plasma (74%) [2]. Therefore, the establishment of a management protocol in cases of MAP is crucial in current obstetrical practice.

Comstock et al. [3] introduced an approach for the detection of MAP in the second and third trimesters of pregnancy by ultrasonography, where the presence of bladder line interruption, absence of the retroplacental clear zone, and presence of placental lacunae were regarded as criteria for the prediction of MAP. This approach was strengthened by criteria that showed a positive predictive value of 48% and a value of 86% with 2 or more criteria. Many reports [4–6] then appeared that assessed the accuracy of a preoperative diagnosis of MAP using ultrasonography. However, there were various advantages and disadvantages concerning the impact of current obstetrical practices on the outcome of MAP predicted by ultrasonography. Shamshirsaz et al. [7] conducted a historical cohort study to investigate the impact of a multidisciplinary protocol on the outcome of MAP compared with a nonmultidisciplinary protocol. In that study, the multidisciplinary protocol was superior to the nonmultidisciplinary protocol in terms of reduction of both emergency surgery and blood loss during the perioperative period, although that cohort study included only confirmed cases of MAP determined by histological examination after delivery. Some reports [8–10] suggested that antenatal diagnosis of MAP by ultrasonography reduced morbidities. On the other hand, it was also reported that suspected cases of MAP before delivery had a poorer outcome and that preoperative diagnosis did not affect the outcome [11, 12]. The reason for deviated results by management with a prediction of MAP seems to be due to a lack of uniformed strategy in terms of diagnosis and treatment. In the future, there is a need to standardize diagnostic criteria and treatment strategy for MAP. Before that, it is still necessary to accumulate information concerning the impact of management with a prediction of MAP by ultrasonography on maternal prognosis.

We conducted a single-arm historical cohort study of women with placenta previa and the absence or presence of risk factors for MAP to evaluate both the accuracy of a prediction of MAP by ultrasonography and the impact of management with a prediction of MAP on maternal prognosis.

## 2. Materials and Methods

We obtained approval (#O-0027) for this study from a constituted Ethics Committee in our institution. We retrospectively examined the medical charts of women having placenta previa and the absence or presence of risk factors for adherent placenta from January 2008 to February 2014 and who were admitted to the Perinatal Center of the University of Miyazaki from January 2008 to February 2014.

**Figure 1:**
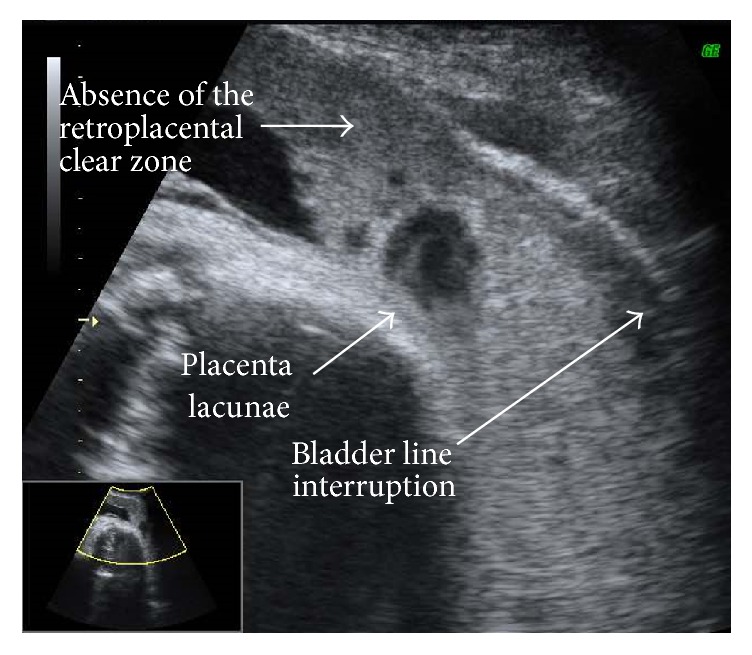
Representative 2D gray scale ultrasound scan for morbidly adherent placenta. Ultrasound scan shows bladder line interruption, absence of the retroplacental clear zone, and placenta lacunae in morbid adherent placenta.

All cases of placenta previa were confirmed by either transabdominal ultrasonography or transvaginal ultrasonography after 20 weeks of gestation. We conducted examinations to detect three ultrasonographic findings regarded as markers for MAP, namely, bladder line interruption, absence of the retroplacental clear zone, and placental lacunae (Figure 1) [3].

Prior to the study period, we performed a preliminary study of 46 cases of placenta previa in an effort to detect MAP by ultrasonography, since MAP was highly suggestive only when all three findings were present, namely, bladder line interruption, absence of the retroplacental clear zone, and placenta lacunae [13]. Women with two findings or less did not have MAP. Thereafter, we proposed cesarean hysterectomy when all three ultrasonography findings were present to strongly suggest MAP.

During the study period, women with placenta previa were managed depending on both risk and the prediction of MAP by ultrasonography. Risk for MAP included history of cesarean delivery, history of uterine curettage, and uterine anomaly. In the group with risks for MAP, women who had all three findings (bladder line interruption, absence of the retroplacental clear zone, and placental lacunae) were regarded as highly suspicious for MAP. In women with two findings or less, removal of the placenta to preserve fertility was attempted. In women without risk for MAP, removal of the placenta was attempted irrespective of the ultrasonographic findings.

Planned operations were performed at 35 to early 37 weeks of gestation. Women whose state was regarded with high suspicion for MAP received combined spinal-epidural anesthesia and then a general anesthesia during hysterectomy. Prior to operations, a bilateral ureteral stent was inserted to prevent ureteral injury during hysterectomy. Additionally, the portio vaginalis was clamped by ring-forceps through the vaginal introitus to allow physicians to recognize the uterine cervix by touch through the abdominal cavity during hysterectomy. When women underwent cesarean section followed by hysterectomy, the placenta was allowed to remain in situ and the uterine cesarean wound was roughly closed. Uterine incisions were made at a site apart from the placenta to avoid unnecessary bleeding. Women having two findings or less received combined spinal-epidural anesthesia and then underwent a low transverse cesarean section followed by an attempt to deliver placenta by gentle traction of the umbilical cord to preserve uterus. If the placenta did not separate, we then attempted a manual removal of placenta. This procedure was based on our previous study, in that women with two ultrasonographic findings or less did not have MAP [13]. In cases of emergencies such as sudden profound bleeding or tocolysis failure before the planned operation, women received general anesthesia and underwent operation without insertion of a bilateral ureteral stent. Access to blood products except for platelets was ensured within 60 minutes following a request. O+ type blood can be given to women in a life-threatening situation.

During the study period, we identified 44 cases of placenta previa. We excluded from the study cases with multifetal pregnancies and deliveries under 22 weeks of gestation. Finally, a total of 41 pregnancies displaying placenta previa were registered in this study. The following characteristics were collected: maternal age, parity (primipara), history of uterine curettage, history of cesarean delivery, and the presence of uterine anomaly. Details of pregnancy outcomes were collected and included gestational age at delivery (weeks), birth weight (g), emergency cesarean delivery, hysterectomy, intraoperative complications such as bladder injury, the number of cases that a blood transfusion was necessary, total blood loss during operation, and postoperative length of maternal stay (days). Confirmation of an adherent placenta was made by histological examination following delivery. Adherent placenta was classified into three severities based on histological examination: placenta accreta, placenta increta, and placenta percreta. We evaluated maternal outcomes according to the cumulative number of ultrasonographic findings in the study group. We then examined the accuracy of prediction for MAP by ultrasonography and the relationship between pathological severity of MAP and the cumulative number of ultrasonographic findings.

Data are expressed as number, incidence (%), mean ± SD, or range. Comparisons between groups were made using Welch's *t*-test. Comparisons among groups were made using the Kruskal-Wallis test or *χ*^2^ tests. Probability values < 0.05 were considered significant. The sensitivity and positive predictive value obtained by utilizing a combination of three findings, comprising bladder line interruption, absence of the retroplacental clear zone, and placental lacunae, with ultrasonography for adherent placenta were evaluated.

## 3. Results

The mean maternal age was 34 ± 5.5 years and gestational age at delivery was 34.1 ± 4.1 weeks. The percentage of nulliparous pregnancies was 22% and the percentage of previous cesarean delivery was 49%. There were also 15 cases having uterine curettage in previous pregnancies and one case of uterine anomaly (subseptate uterus) (Table 1).

In women with risk for MAP (*n* = 28), nine with three findings comprising bladder line interruption, absence of the retroplacental clear zone, and placental lacunae were regarded as highly suspicious for MAP and underwent cesarean section followed by hysterectomy. Eventually, nine women were diagnosed with MAP following histological examination. Five women having two findings underwent removal of the placenta. Two of the five women subsequently underwent hysterectomy due to bleeding, and placenta increta and accreta were confirmed in these two women following histological examination. In the remaining three with two findings, we observed one case of MAP (placenta accreta) following histological examination of placenta, in that a part of myometrium tissue was observed in placenta. Fourteen women having one finding or less underwent removal of the placenta. One of the fourteen women subsequently underwent hysterectomy due to difficulty of placental removal, and MAP (placenta accreta) was confirmed in this case following histological examination. Finally, we preserved 16 of 28 fertilities in women with risk for MAP. Fourteen cases of MAP were observed by histological examination following delivery. The sensitivity and positive predictive value for the detection of MAP by ultrasonography were 64% and 100%, respectively. The pathological severity of MAP was significantly correlated with the cumulative number of ultrasonographic findings (*p* < 0.01). Eight out of nine women having three findings showed placenta percreta or increta. In contrast, only one case of placenta increta was found in 19 women having two findings or less (Table 2). In women without risk for MAP (*n* = 13), there were no cases of hysterectomy or MAP (Table 2).

Analysis of maternal complications indicated a significant difference of blood loss among groups including women without risk (*p* = 0.02). One case resulted in massive blood loss (13,310 ml) following hysterectomy. This case had two findings composed of bladder line interruption and placenta lacunae and involved an emergency cesarean section due to uncontrollable bleeding followed by removal of the placenta. Except for the group with two findings, including the aforementioned massive bleeding case, the mean blood loss was 2186 ml or less in the other study groups. There was no difference of blood loss between women that were highly suspicious for MAP and women without risk (2186 ± 1438 ml versus 1656 ± 848 ml, resp.; *p* = 0.34) (Table 2). The incidence of blood transfusion did not differ among the groups including women without risk (*p* = 0.06). Additionally, the incidence of emergency cesarean section did not differ among the groups including women without risk (*p* = 0.58). Bladder injury only occurred in one of the women highly suspicious for MAP. The case was treated by surgical repair followed by an uncomplicated postoperative course. There was no difference of postoperative hospital stay among the groups including women without risk (*p* = 0.26).

## 4. Discussion

We have shown high sensitivity (64%) and a high positive predictive value (100%) for the detection of MAP by ultrasonography in women with risk (*n* = 28). The pathological severity of MAP was significantly correlated with the cumulative number of ultrasonographic findings. Additionally, cesarean hysterectomies were safely employed in cases regarded with high suspicion for MAP (*n* = 9) without profound blood loss. We preserved 16 of 28 fertilities in women with risk. There were no cases of MAP among women without risk. Thus, management is dependent on risk, and the prediction of MAP by ultrasonography is beneficial to both physicians and women in helping to obtain tolerable maternal outcomes.

According to a report by Comstock et al. [3], the sensitivity and positive predictive value for the detection for MAP by ultrasonography with any finding, such as the presence of bladder line interruption, absence of the retroplacental clear zone, or presence of placental lacunae, at 15 to 40 weeks of gestation were 100% and 48%, respectively. In order to obtain a high predictive value for the detection of adherent placenta, a combination of multiple findings may be superior, although this approach may not be very sensitive. According to Comstock et al. [3], a combination of two or more findings showed 80% sensitivity and a positive predictive value of 86%. Use of a combination of smallest sagittal myometrium thickness, lacunae, and bridging vessels, in addition to the number of cesarean sections and placental location, has also been reported to be useful for predicting MAP. As a consequence, a score of 0–9 representing the Placenta Accreta Index (PAI score) was created for predicting MAP [5]. A high PAI score indicates a high positive predictive value, but with low sensitivity. Bowman et al. [14] suggested that the prediction of MAP by ultrasonography may not be very sensitive. However, our study reveals a high positive predictive value (100%) when using a combination of multiple findings without loss in sensitivity (64%). In women without risk for MAP, there were no cases of hysterectomy or MAP. Therefore, our management was dependent on risk and the prediction of MAP by ultrasonography to provide high sensitivity and a high positive predictive value for the detection for MAP, which reduced unnecessary hysterectomies.

The ultimate management goal for MAP is to minimize mortality and morbidity in affected women. Therefore, we need to obtain more effective treatment protocol including prenatal diagnosis of MAP and procedures. It has been suggested that suspected cases prior to delivery result in poorer outcomes because more cases comprising clinically significant morbidity may be included [11]. It was also reported that preoperative diagnosis of MAP did not affect the outcome [12]. In contrast, our prediction of MAP showed beneficial effect to women in helping to obtain tolerable maternal outcomes and provided similar conclusion like previous reports [8–10]. Those suggest that antenatal diagnosis of MAP by ultrasonography reduces maternal morbidity. In terms of minimizing blood loss, a multidisciplinary approach and planned operations are preferable [15]. Shamshirsaz et al. [7] conducted a historical cohort study to investigate the impact of multidisciplinary protocols on the outcome of placenta accreta and indicated that the multidisciplinary protocol was superior to the nonmultidisciplinary protocol because both emergency surgery and blood loss at the perioperative period were reduced. Their protocol and management in the operation room were similar to our protocol except for the operating procedures (modified radical hysterectomy with or without intraoperative arterial embolization). In our study, the portio vaginalis was clamped by ring-forceps through the vaginal introitus to allow physicians to recognize the uterine cervix by touch through the abdominal cavity during the operation. Employing a combination of techniques comprising clamping of the portio vaginalis and use of a bilateral ureteral stent allowed us to conduct simple hysterectomies more easily without ureteral injury. There was only one case of bladder injury and no cases of ureteral injury in highly suspicious cases of MAP. Thus, our surgical procedure also achieved permissible morbidity. Additionally, we were able to make a judgment concerning the preservation of fertility among cases regarded with moderate to low suspicion (16 of 28 fertilities preserved). These results provide an explanation of the morbidity associated with MAP prior to operations and highlight the merits of our study.

We experienced one case with two findings comprising bladder line interruption and placenta lacunae, resulting in massive bleeding (13,310 ml). Prior to the current study, we performed a preliminary study of 46 cases of placenta previa in an effort to detect MAP by ultrasonography, in that women with two findings (bladder line interruption and placenta lacunae) did not have MAP. The MAP was only confirmed when all three findings were present [13]. Therefore, we proposed manual removal of placenta in order to preserve fertility when two ultrasonography findings were present. In current study, the remaining four with two findings (retroplacental clear zone and placental lacunae) did not have severe MAP (i.e., increta or percreta), followed by preservation of fertility (Table 2). Except for the aforementioned case, the pathological severity of MAP was significantly correlated with the cumulative number of findings. According to the literature, the findings of bladder line interruption have a high positive predictive value for MAP [6, 16]. In addition, the extirpative method is strongly deprecated, because it is associated with significant hemorrhagic morbidity [17]. Therefore, we have to reconsider our treatment protocol when women have two findings comprising bladder line interruption and others.

There are some limitations in our study. Firstly, since our study was not a comparative study, we were unable to show the superiority of our protocol compared with that of others. Our protocol did not include catheter intervention for reducing blood loss during operation. Currently, the management by multidisciplinary team involving interventional radiologist seems to be more effective for reducing maternal morbidity [7, 15, 18]. We need a subsequent examination to investigate the impact of catheter intervention during operation on our protocol. Secondly, since our investigations comprised a single institutional study, it is unclear whether similar outcomes would be obtained in other tertiary facilities using the same protocol.

In conclusion, under circumstances involving varied information concerning the advantages and disadvantages of prediction by ultrasonography in terms of maternal outcome, we demonstrated the usefulness of a management protocol based on both the risk and prediction of MAP by three ultrasonographic markers for MAP; those are bladder line interruption, absence of the retroplacental clear zone, and placental lacunae. Employment of a multidisciplinary approach by further examinations for the detection of MAP and introducing effective procedures to reduce blood loss is desirable to achieve permissible morbidity.

## Figures and Tables

**Table 1:** Demographic data of the study group. Results are expressed as number, mean ± SD, or incidence (%).

Maternal age (years)	34.0 ± 5.5
Primipara	9 (21.9)
Gestational age at delivery (weeks)	34.1 ± 4.1
History of caesarean delivery	20 (48.8)
1	16 (39.0)
2	3 (7.3)
≥3	1 (2.4)
History of uterine curettage	15 (36.6)
Uterine anomaly	1 (2.4)

Results are expressed as number, mean ± SD, or incidence (%).

**Table 2:** Maternal outcomes according to ultrasonographic findings. Results are expressed as number, mean ± SD, or incidence (%). Comparisons between groups were made using Welch's *t*-test. Comparisons among groups were made using the Kruskal-Wallis test or *χ*^2^ tests. NS: not significant.

	With risk			Without risk	*p*
Cumulative number of ultrasonographic findings	0~1	2	3	0~1	

*n*	14	5	9	13	

Absence of the retroplacental clear zone	0	4	9	0	
Bladder line interruption	0	1	9	0	
Placenta lacunae	12	5	9	5	

Emergency cesarean section	8	3	3	3	0.58
Hysterectomy	1 (7%)	2 (40%)	9 (100%)	0	<0.01
Total blood loss (ml)	1154 ± 800	4376 ± 5051	2186 ± 1438	1656 ± 848	0.02
Blood transfusion	6 (43%)	3 (60%)	5 (56%)	1 (8%)	0.06
Bladder injury	0	0	1	0	NS
Postoperative hospital stay (days)	8 (6–12)	9 (7–11)	10 (7–17)	8 (7–14)	0.26
Confirmed MAP by histological study	2	3	9	0	
(increta or percreta)	(0)	(1)	(8)		0.03

Results are expressed as number, incidence (%), mean ± SD, or range.
